# LNK deficiency decreases obesity-induced insulin resistance by regulating GLUT4 through the PI3K-Akt-AS160 pathway in adipose tissue

**DOI:** 10.18632/aging.103658

**Published:** 2020-09-10

**Authors:** Xiaozhu Zhong, Chuanfeng Ke, Zhaoxi Cai, Hao Wu, Yang Ye, Xiaolin Liang, Liqun Yu, Sushi Jiang, Jun Shen, Laiyou Wang, Meiqing Xie, Guanlei Wang, Xiaomiao Zhao

**Affiliations:** 1 Department of Obstetrics and Gynecology, Sun Yat-Sen Memorial Hospital, Sun Yat-Sen University, Guangzhou 510120, China; 2 Department of Gastrointestinal Surgery, The First Affiliated Hospital of Guangzhou Medical University, Guangzhou 510120, China; 3 Department of Radiology, Sun Yat-Sen Memorial Hospital, Sun Yat-Sen University, Guangzhou 510120, China; 4 Department of Pharmacology, Guangdong Pharmaceutical University, Guangzhou 510006, China; 5 Department of Gynecology, Aviation General Hospital, Beijing 100012, China; 6 Department of Clinical Pharmacy, Guangdong Provincial People’s Hospital, Guangdong Academy of Medical Sciences, Guangzhou 510080, China; 7 Department of Pharmacology, Zhongshan School of Medicine, Sun Yat-Sen University, Guangzhou 510080, China

**Keywords:** LNK, obesity, insulin resistance, glucose translocation

## Abstract

In recent years, LNK, an adapter protein, has been found to be associated with metabolic diseases, including hypertension and diabetes. We found that the expression of LNK in human adipose tissue was positively correlated with serum glucose and insulin in obese people. We examined the role of LNK in insulin resistance and systemic energy metabolism using LNK-deficient mice (*LNK^-/-^*). With consumption of a high-fat diet, wild type (WT) mice accumulated more intrahepatic triglyceride, higher serum triglyceride (TG), free fatty acid (FFA) and high sensitivity C-reactive protein (hsCRP) compared with *LNK^-/-^* mice. However, there was no significant difference between *LNK^-/-^* and WT mice under normal chow diet. Meanwhile, glucose transporter 4 (GLUT4) expression in adipose tissue and insulin-stimulated glucose uptake in adipocytes were increased in *LNK^-/-^* mice. *LNK^-/-^* adipose tissue showed activated reactivity for IRS1/PI3K/Akt/AS160 signaling, and administration of a PI3K inhibitor impaired glucose uptake. In conclusion, LNK plays a pivotal role in adipose glucose transport by regulating insulin-mediated IRS1/PI3K/Akt/AS160 signaling.

## INTRODUCTION

Metabolic syndrome, also known as insulin resistance syndrome, is currently a common metabolic disorder with the increasing prevalence of obesity [1]. Obesity-associated insulin resistance in insulin-target tissues including the liver, skeletal muscle and adipose tissue is an early clinical feature of the development of Type 2 diabetes [2]. Thus, it is necessary to discover satisfactory biomarkers for the diagnosis of insulin resistance (IR). Adipose tissue is crucial in regulating insulin sensitivity and the risk for diabetes through its lipid storage capacity as well as thermogenic and endocrine functions. Insulin resistance in adipose tissue is the inability of insulin to activate adipose glucose transport, promote lipid uptake, and suppress lipolysis. While decreased adipose glucose uptake is demonstrated in both *in vivo* and *in vitro* models, the mechanism and metabolic impact of impaired insulin-mediated glucose uptake in adipose tissue is unclear [3].

Obesity and insulin resistance are associated with reduced PI3K activity in adipose tissue. The phosphatidylinositol 3-kinase (PI3K)/Akt pathway is essential for insulin-regulated glucose metabolism. The regulation of glucose transport and use by insulin is central to the maintenance of whole-body glucose homeostasis [4]. The binding of insulin to the extracellular domain of the insulin receptor (IR) leads to phosphorylation and a conformational change in its intracellular tyrosine kinase domain that allows the IR to serve as a signaling scaffold and docking site for many proteins, including IR substrate (IRS) proteins. Activation of PI3K and Akt plays a critical role in the regulation of insulin-stimulated glucose uptake by promoting the translocation of GLUT4 [5]. Akt substrate of 160 kDa (AS160), also called TBC1D4, becomes phosphorylated in insulin-stimulated 3T3-L1 adipocytes; this is important for insulin-stimulated GLUT4 translocation and glucose transport [6].

LNK (also called SH2B3) is a member of the SH2B family of adaptor proteins that influences a variety of signaling pathways mediated by Janus kinase and receptor tyrosine kinases. Biochemical and imaging techniques showed that the bulk of LNK remains in the cytoplasm, particularly in the perinuclear region, while a small portion of the protein is localized in the plasma membrane [7, 8]. Although LNK is mainly expressed in hematopoietic, neuronal and endothelial cells, SH2B proteins have been shown to function during multiple physiological processes including glucose homeostasis, energy metabolism and hematopoiesis [9–11]. SH2B3 784T>C variant could contribute to the pathogenesis of Type 1 diabetes through impaired immune response that promotes the activation and expansion of self-reactive lymphocytes in susceptible individuals [12]. It has been proven that the SH2 domain of adaptor proteins can bind to insulin receptors in response to insulin [11]. As an important regulator of insulin and insulin signaling in drosophila, LNK (mammalian homologue of drosophila LNK is considered as SH2B1) may play a role in the development of insulin resistance [13]. A comprehensive understanding of the pathogenesis involved in insulin resistance may enable the identification of targets for improving insulin sensitivity as well as preventing and treating Type 2 diabetes (T2D). We previously found that LNK inhibited the PI3K-Akt and MAPK-ERK signaling response to insulin, and LNK probably played a role in the development of insulin resistance [14]. Meanwhile, LNK might be involved in adipose tissue dysfunction [15]. In this study, we aim to use *LNK^-/-^* mice as a model to assess the effects of LNK on insulin resistance.

## RESULTS

### Expression of LNK in adipose tissue and correlations with serum glucose and insulin in obese people

Seventy-two patients were included from January 1, 2017 to June 30, 2018. Adipose tissue samples were obtained from 31 patients who received laparoscopic surgery for fallopian tube or pelvic disorders in the Gynecologic department of Sun Yat-sen Memorial Hospital of Sun Yat-sen University, and 41 patients received abdominal surgery for gastrointestinal tumors or extreme obesity in the First Affiliated Hospital of Guangzhou Medical University. Ages of patients ranged from 21 to 65 years old. Body mass index (BMI) of patients ranged from 16.40 to 36.36 kg/m^2^.

**Figure 1:**
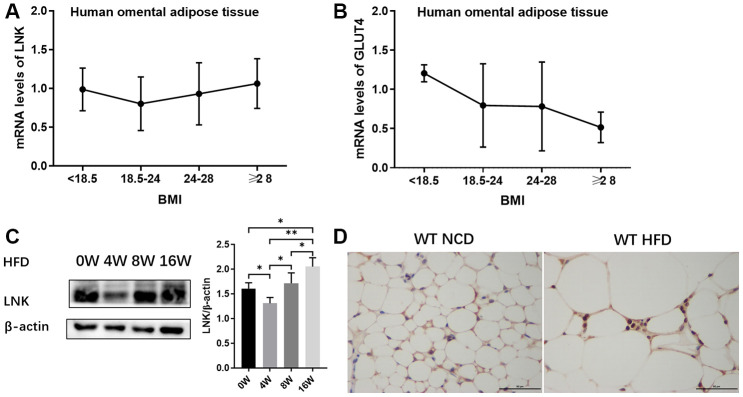
**Expression of LNK in adipose tissue.** The mRNA expression of LNK (**A**) and GLUT4 (**B**) in human omental adipose tissue (n=72). The representative image (**C**) of LNK protein expression in adipose tissue of mice with HFD feeding for 0, 4, 8 and 16 weeks were shown (n=6 mice per group). Localization of LNK in adipose tissue of wild type mice with both normal chow diet and high-fat diet (**D**). Data were shown as mean ± SD.

The relationships between the expression of LNK in human omental adipose tissue and metabolic features are shown in Figure 1 and Table 1. LNK showed a decreasing trend with the elevation of BMI and then an increasing trend after a BMI level of 24-28 kg/m^2^ (Figure 1A). The mRNA expression of GLUT4 in human omental adipose tissue showed a downward trend with an elevated BMI (Figure 1B). Expression of LNK was positively correlated to fasting serum glucose (r=0.745, *P*<0.05), 2-h serum glucose (r=0.924, *P*<0.05) and fasting serum insulin (r=0.940, *P*<0.05) in the group of patients with BMI ≥28 kg/m^2^ (Table 1). The expression of LNK in mice visceral adipose tissue decreased after 4 weeks of high-fat diet and subsequently increased after 8 weeks of high-fat diet (Figure 1C). Consistent with this change, LNK relieved impaired glucose tolerance in HFD feeding mice for 4 weeks (Supplementary Figure 2) Immunohistochemistry showed that LNK was mainly expressed in the cytoplasm and a small part of the protein was localized in the plasma membrane of mice adipocytes (Figure 1D).

**Table 1:** Correlation between LNK expression in adipose tissue and metabolic features in patients.

		**BMI**	**mRNA level of GLUT4**	**systolic pressure**	**waistline**	**WHR**	**GLU 0h**	**GLU 2h**	**INS 0h**
Total population mRNA level of LNK n=72	r	.239*	.224	.041	.278*	.258*	.242	.516*	.541*
	P	.043	.121	.753	.026	.040	.059	.024	.014
BMI<18.5 kg/m^2^ mRNA level of LNK n=7	r	-.030	.235	.731	-.054	-.682	-.477	-	-
	P	.949	.849	.269	.919	.135	.416	-	-
BMI 18.5~24 kg/m^2^ mRNA level of LNK n=34	r	.250	0.073	-.359*	.382*	.370*	.569**	-.395	-.278
	P	.141	0.755	.047	.031	.037	.001	.333	.436
BMI 24~28 kg/m^2^ mRNA level of LNK n=20	r	.061	.448	.055	.135	-.089	.107	.574	.611
	P	.794	.082	.830	.582	.716	.683	.520	.581
BMI ≥28 kg/m^2^ mRNA level of LNK n=11	r	.139	-.019	-.002	.398	.383	.745*	.924*	.940*
	P	.684	.962	.996	.254	.274	.013	.025	.018

BMI, body mass index; GLU, glucose; GLUT4, glucose transporter 4; INS, insulin; WHR, waist to hip ratio.

*p < 0.05, **p < 0.01.

### LNK influences glucose and lipid metabolism in the *in vivo* model of diet-induced obesity (DIO) mice

**Figure 2:**
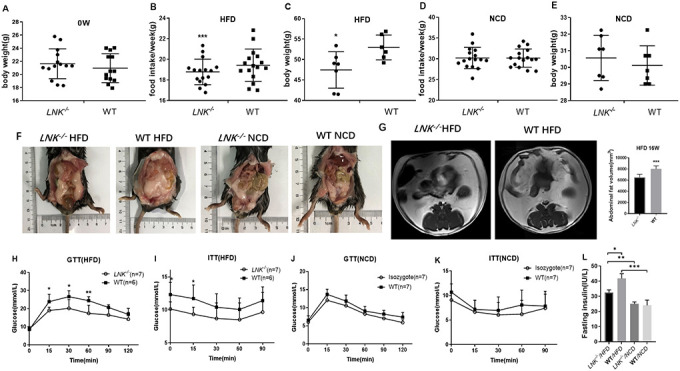
**LNK resulted in worse diet-induced obesity, impaired glucose tolerance and increased insulin resistance under HFD feeding for 16 weeks.** Body weights (**A**) were measured in 6 to 8-week-old *LNK^-/-^* (n=13) or WT mice (n=13). (**B**–**E**) Food intake and body weight after HFD or NCD feeding for 16 weeks were shown. Abdominal pathologic specimen (**F**) and MRI analyses (**G**) were shown to compare abdominal fat volume. GTTs (**H** and **J**) and ITTs (**I** and **K**) in WT (filled symbols) or *LNK^-/-^* mice (open symbols) were measured. Blood glucose concentrations were measured at the indicated times. Fasting insulin concentration (**L**) were measured in mice after NCD or HFD feeding for 16 weeks. HFD: *LNK^-/-^* n=7, WT n=6; NCD: *LNK^-/-^* n=6, WT n=7. Data were shown as mean ± SD. Statistical analysis was performed by Student’s *t*-test. *p < 0.05, **p < 0.01, ***p < 0.001.

An *in vivo* DIO mice model was successfully built by detecting the body weight, blood glucose level in GTT as well as ITT, and MRI of the abdominal fat tissue volume. There were significant differences in food intake (Figure 2B) and body weight (Figure 2C) between *LNK^-/-^* and WT mice in HFD group (*P*<0.05). The abdominal anatomy of the mice under high-fat diet and normal chow diet (Figure 2F) were shown. VAT volume of HFD-fed *LNK^-/-^* mice was significantly higher than that of WT mice (Figure 2G).

Given that LNK-deletion promoted an insulin-resistant phenotype, we then wanted to examine whether glucose and lipid metabolism were altered in these mice. Compared with *LNK^-/-^* mice, blood glucose levels at 15, 30, 60 min of GTTs and 0, 15 min of ITTs were higher in WT mice after 16-week HFD (*P*<0.05, Figure 2H and 2I). The fasting insulin levels (32.58±1.64 μIU/mL *vs*. 41.76±3.24 μIU/mL, *P*<0.05, Figure 2L) were significantly increased after 16-week HFD in WT mice. There were no significant differences in food intake, body weight, VAT volume, GTTs, ITTs and fasting insulin level in WT and *LNK^-/-^* mice with standard chow diet (*P*>0.05, Figure 2D, 2E, 2J, 2K and 2L).

**Figure 3:**
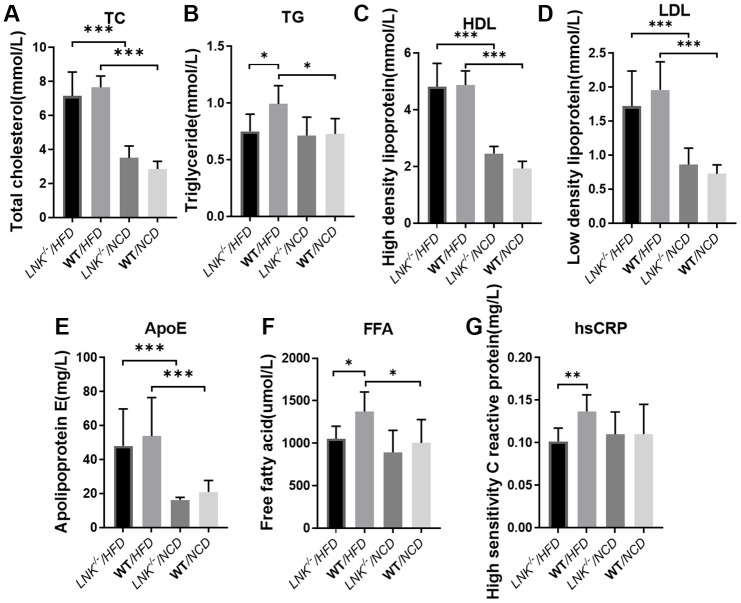
**LNK deficiency influences adipose metabolism and systemic inflammation.** Fasting total cholesterol (**A**), triglyceride (**B**), HDL (**C**), LDL (**D**), apolipoprotein (**E**), free fatty acid (**F**), high-sensitivity c-reactive protein (**G**) levels in mice serum at week 16 on the HFD or NCD feeding. HFD: *LNK^-/-^* n=7, WT n=6; NCD: *LNK^-/-^* n=6, WT n=7. Bars indicate mean ± SD. Statistical analysis was performed by One-way analysis of variance (ANOVA). *p < 0.05, **p < 0.01, ***p < 0.001.

Serum lipid and liver enzyme levels were examined in both the HFD and NCD groups. The levels of fasting triglyceride (0.99 ± 0.16 mmol/L *vs*. 0.75 ± 0.15 mmol/L, *P*<0.05, Figure 3B), free fatty acids (1373.67 ± 229.25 μmol/L *vs*. 1051.71 ± 148.00 μmol/L, *P*<0.05, Figure 3F) and high sensitivity C reactive protein (0.14±0.02 mg/L *vs.* 0.10±0.02 mg/L, *P*<0.01, Figure 3G) were elevated in WT mice relative to *LNK^-/-^* mice in the HFD group. Total cholesterol (Figure 3A), HDL (Figure 3C), LDL (Figure 3D), and ApoE (Figure 3E) levels were not significantly different between *LNK^-/-^* and WT mice after 16-week HFD. However, these serum biochemical indexes were not different between *LNK^-/-^* and WT mice which were fed a standard chow diet. These data implied that the presence of LNK may impair glucose and lipid metabolism under a circumstance of obesity-induced insulin resistance.

### LNK knockout mice show ameliorative hepatic steatosis

**Figure 4:**
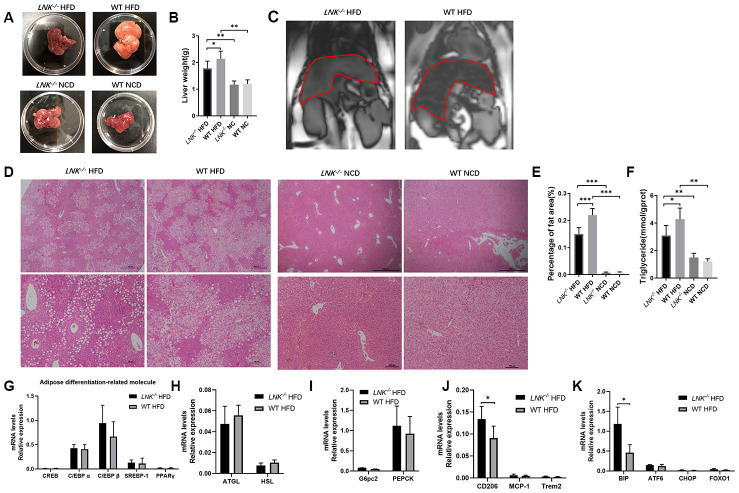
**LNK deficiency relieves hepatic steatosis.** Representative images of pathologic specimen (**A**) of liver tissue were shown. Liver weight (**B**) was compared in WT or *LNK^-/-^* mice after HFD or NCD feeding for 16 weeks. Out-of-phase MRI for diagnosis of fatty liver of mice with HFD (**C**) and representative images of H&E staining of the liver (**D**, HFD: *LNK^-/-^* n=7, WT n=6; NCD: *LNK^-/-^* n=6, WT n=7. 10 images were quantified per mouse) were shown. Scale bars: 500 μm, 100 μm and 50 μm. Percentage of fat area in liver pathological tissue slice (**E**) and liver triglycerides (**F**) were shown. The mRNA expression of adipose differentiation-related molecule (**G**), lipolysis-related molecule (**H**), gluconeogenesis-related molecule (**I**), inflammation-related molecule (**J**) and endoplasmic reticulum stress-related molecule (**K**) in liver tissue of mice fed with HFD after 16 weeks were compared. HFD: *LNK^-/-^* n=7, WT n=6; NCD: *LNK^-/-^* n=6, WT n=7. Data were shown as mean ± SD. Statistical analysis was performed by ANOVA (**B**, **C**, **E** and **F**) and Student’s *t*-test (G to K). *p < 0.05, **p < 0.01, ***p < 0.001.

HFD-induced obesity can lead to hepatosteatosis, and we found that the deletion of LNK alleviated the hepatic lipid profile in HFD mice (Figure 4). The anatomy and out-of-phase MRI of the liver showed increased lipid deposition in WT mice (Figure 4A–4C). Histological analysis of the liver showed that the severity of hepatic steatosis in the liver was increased with HFD; and WT mice developed more severe steatosis than *LNK^-/-^* mice, with increased hepatic triglycerides (Figure 4D–4F). Markers of adipose differentiation (Figure 4G), lipolysis (Figure 4H) and gluconeogenesis (Figure 4I) were not different between genotypes. CD206 and binding immunoglobulin protein (BIP) were decreased in the livers of *LNK^-/-^* HFD mice (Figure 4J, 4K), which indicated a reduction in inflammation and endoplasmic reticulum stress.

### LNK influenced glucose translocation by regulating insulin signaling

**Figure 5:**
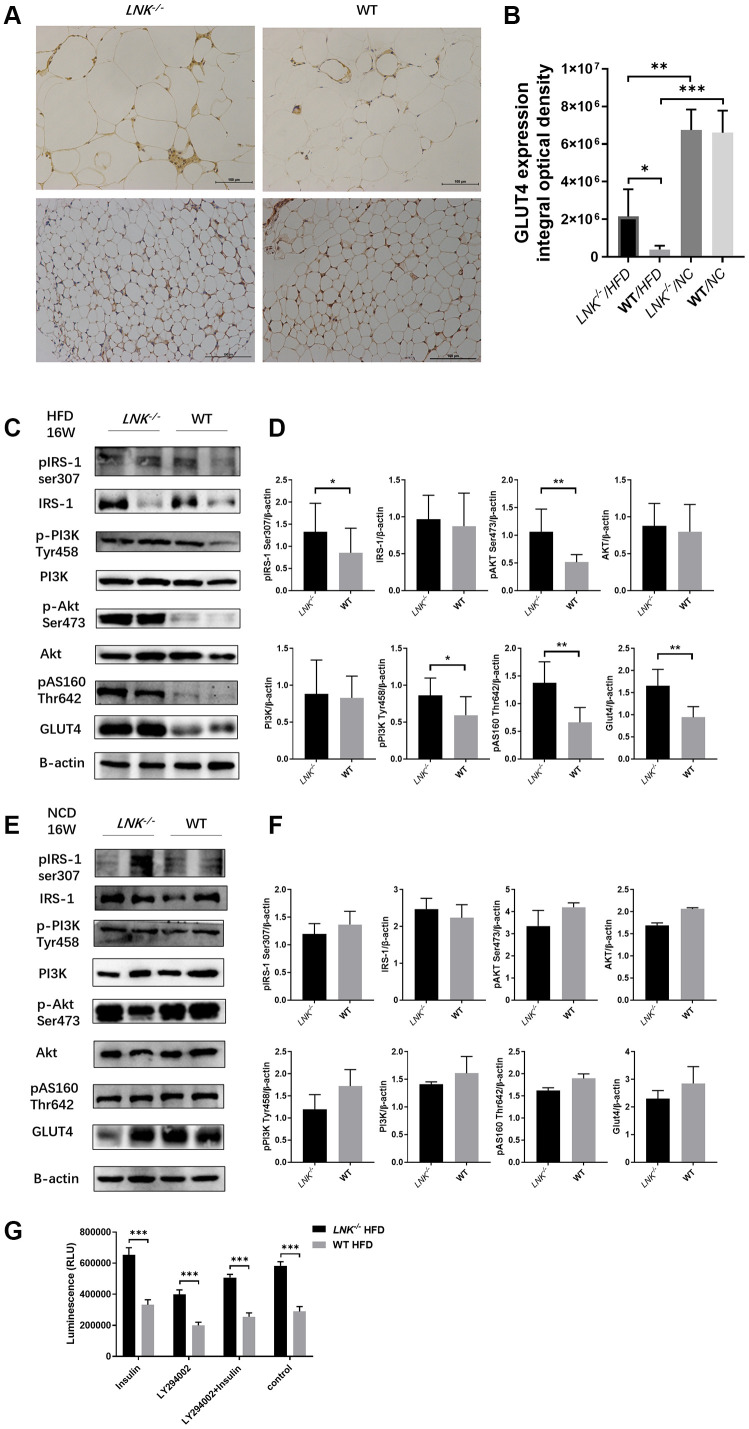
**LNK deficiency altered insulin signaling and the expression of GLUT-4 protein in adipose tissues in obesity-induced insulin resistance model.** (**A**, **B**) Representative images of immunohistochemical stain of GLUT4 in adipose tissue were shown (HFD: *LNK^-/-^* n=7, WT n=6; NCD: *LNK^-/-^* n=6, WT n=7. Five images were quantified per mouse). (**C**, **D**) LNK impairs IRS1 serine phosphorylation, PI3K tyrosine phosphorylation, Akt serine phosphorylation and AS160 threonine phosphorylation as shown by WB in HFD-fed mice visceral adipose tissue (*LNK^-/-^* n=7, WT n=6 with three replicates. Representative images were shown). (**E**, **F**) GLUT4 expression and IRS1 serine phosphorylation, PI3K tyrosine phosphorylation, Akt serine phosphorylation and AS160 threonine phosphorylation as shown by WB in NCD-fed mice adipose tissue (*LNK^-/-^* n=6, WT n=7 with three replicates. Representative images were shown). (**G**) LNK deficiency improves glucose uptake in primary adipocyte from mice after HFD feeding for 16 weeks (n=6 per group). The primary adipocytes were differentiated into mature adipocytes and were then stimulated by LY294002 (50 μM) and insulin (10 μM) for 30 min or insulin (10 μM) for 30 min alone. Scale bars: 100 μm. Data were shown as mean ± SD. Statistical analysis was performed by ANOVA (**B** and **G**) and Student’s *t*-test (**D** and **F**). *p < 0.05, **p < 0.01, ***p < 0.001.

Since insulin regulates glucose transporter GLUT4 in adipose and muscle tissues, it seemed probable that adipose GLUT4 levels were elevated in *LNK^-/-^* mice. To test this hypothesis, we measured adipose GLUT4 by WB and IHC, and found that high-fat diet-fed *LNK^-/-^* mice showed marked improvement in glucose transport, however on the chow diet, LNK deficiency did not significantly alter glucose transport (Figure 5A–5C). Furthermore, we found that the insulin signaling pathway was involved. Levels of IRS1 serine phosphorylation, PI3K tyrosine phosphorylation, Akt serine phosphorylation and AS160 threonine phosphorylation adipose tissue decreased in wild-type mice compared with *LNK^-/-^* mice after 16-weeks of HFD. There were no significant differences in the phosphorylation levels of insulin signaling molecules between WT and *LNK^-/-^* mice with standard chow diet (Figure 5E, 5F). Insulin-stimulated glucose uptake in primary adipocytes was significantly improved with LNK knockout. *Ex vivo* administration of 50 μM LY294002, an inhibitor of phosphatidylinositol 3-kinase, attenuated the insulin-induced glucose uptake in adipocytes derived from VAT of *LNK^-/-^* mice (Figure 5G).

## DISCUSSION

In this study, we aimed to unravel the molecular mechanisms by which LNK controls glucolipid metabolism and obesity-induced insulin resistance. Deciphering the role of LNK and its contribution to the pathogenesis of obesity-induced insulin resistance is of importance since it may be a new potential target for innovative therapeutic strategies. The work presented here provides new insights into LNK function in adipose tissue.

Insulin resistance is a state in which peripheral tissue demonstrates impaired glucose uptake in response to insulin, which is an early predictor of Type 2 diabetes and is highly correlated with obesity [2]. Weight reduction often leads to increased insulin sensitivity in peripheral tissue [16]. The mechanism by which obesity may lead to insulin resistance is still unknown. In this study, we found that the expression of LNK in human adipose tissue is positively correlated with serum glucose and insulin in the obese group, so we hypothesize that LNK was involved in glucolipid metabolism and may contribute to alterations in glucose homeostasis and lipid metabolism associated with obesity-induced insulin resistance.

A major piece of evidence for this new role of LNK in insulin resistance is derived from our *LNK^-/-^* mouse studies. We found that LNK deficiency reduced the appetite of mice for high-fat diet. Food intake is remarkably complex, as it is influenced by diverse endocrinology and multiple interacting neural circuits. Factors which influence appetite include genes, epigenetics, hormones, other metabolic influences, as well as neural, psychological, behavioral and emotional characteristics [17]. Food intake can be considered as an integrated response over a prolonged period of time that maintains the levels of energy stored in adipocytes. The efficacy of satiation signals is modified by adiposity via signals such as insulin and leptin that act in the hypothalamic ARC and other areas, and these signals are in turn modified by adiposity hormones that indicate the fat content of the body [18]. It has been reported that *LNK^-/-^* mice demonstrated spontaneous villi atrophy and T cell mediated inflammation in the small intestine [19]. Therefore, LNK may cause self-regulation of appetite by affecting the neuroendocrine system and brain-gut axis in the context of obesity.

Adipose tissue plays a critical role in obesity-associated metabolic complications of insulin resistance, and adipocyte dysfunction promotes fatty acid esterification and hepatic triglyceride synthesis, exacerbating hepatic steatosis, hepatic insulin resistance, and hypertriglyceridemia [3]. We explored the role of LNK in adipose tissue and found that the loss of function of LNK relieved the detrimental metabolic effects of high-fat feeding in mice, including the content of visceral fat, glucose tolerance and fatty degeneration of liver. In response to a high-fat diet, WT mice developed higher serum levels of TG, FFA, and hsCRP compared with *LNK^-/-^* mice. Serum CRP reflects the level of systemic inflammation. Free-fatty acids (FFAs) are well-characterized factors which induce the production of inflammatory factors and insulin resistance in adipocytes [20]. Armoni et al. discovered that Type 2 diabetes and obesity were associated with the impaired regulation of GLUT4 gene expression and elevated levels of free fatty acids and pro-inflammatory factors [21].

Hepatic fat accumulation has been linked to insulin resistance and the prevalence of non-alcoholic fatty liver disease (NAFLD) increases with obesity [22]. Our results indicate that the role of LNK in adipocytes is vital for glucolipid metabolism, and is associated with obesity-induced NAFLD and insulin resistance. Evidence showed that white adipose tissue dysfunction promoted NAFLD [23]. NAFLD may result primarily from increased *de novo* hepatic lipogenesis or secondarily from adipose tissue lipolysis [24]. The presence of lipogenic transcription factors, steatosis, endoplasmic reticulum stress, oxidative stress and inflammatory mediators have been implicated in the alterations of nuclear factors in NAFLD [25]. However, it remains unclear on what role the hepatic lipid droplets and associated proteins play in promoting the disease process. We examined the change of adipose differentiation, lipolysis, gluconeogenesis, inflammation and endoplasmic reticulum stress-related molecule. Our results indicate that inflammation and endoplasmic reticulum stress may play a role in the development of NAFLD under the circumstance of LNK deficiency.

Insulin plays an essential role in mammalian metabolic homeostasis by accelerating the disposal of postprandial glucose into muscle and adipose tissue. This step is mediated via the insulin-regulated translocation of GLUT4 from intracellular vesicles to the plasma membrane [26]. Interestingly, GLUT4 protein was markedly reduced in adipose cells from individuals with T2D and in around 30% of individuals with a genetic predisposition for T2D long before T2D develops [27, 28]. It should be emphasized that the ability of adipose tissue to regulate whole-body insulin sensitivity is not a consequence of its capacity to take up glucose on insulin stimulation, as it only accounts for around 10% of the glucose load [29]. However, glucose uptake is crucial for normal adipose tissue function. Genetic deletion of GLUT4 from adipose tissue produces a similar degree of whole-body insulin resistance in mice as does the deletion of GLUT4 from skeletal muscle, the tissue responsible for most of insulin-stimulated glucose uptake [30]. It is important to understand the role of GLUT4/glucose metabolism in regulating adipose tissue biosynthesis and the secretion of lipids and other adipokines, which has profound effects on whole-body insulin sensitivity. We found that LNK inhibition increased GLUT4 translocation in adipose tissue. *In vivo* glucose transport into adipose tissue of *LNK^-/-^* mice was markedly improved compared with WT mice.

Given the magnitude of the effects of LNK, we conducted detailed *in vivo* studies to elucidate the mechanisms of LNK-induced insulin resistance. *LNK* mutations affecting the SH2 domain influenced a variety of signaling pathways [8, 31–33]. LNK regulated the PI3-kinase as reflected by Akt phosphorylation [34]. With respect to more in-depth mechanisms, we found that LNK led to decreased insulin-stimulated IRS1 serine phosphorylation, PI3K tyrosine phosphorylation, Akt serine phosphorylation, AS160 threonine phosphorylation and GLUT4 expression. Based on these studies, we suggest that intracellular LNK could lead to the inhibition of IRS1 serine phosphorylation and downstream signaling. Here, we provided evidence that LNK was a regulator of the insulin signaling pathway and a major cause of impaired glucose metabolism, the defining feature of insulin resistance.

Taizo et al. also examined the effects of LNK knockout on glucose tolerance [35]. They reported that LNK reduced the risk of developing diabetes via the regulation of adipose inflammation. In their studies, LNK deficiency resulted in impaired glucose tolerance and increased insulin resistance under normal feeding conditions, but this was not observed in our *LNK^−/−^*mice. Besides, they fed the mice with HFD for 10 weeks, while we carried out 16 weeks of HFD in our study. Although the results were different, we both found that LNK was associated with adipose dysfunction and IR. Our findings suggested that abnormal glucose transport was more important, and they found that LNK also affected adipose inflammation. This was most likely due to different genetic backgrounds and different experimental conditions in knockout mice. In addition, the environment of the mice colony may contribute to the difference. Their mice were maintained under specific pathogen–free (SPF) housing conditions, while we bred the mice under SPF conditions and operated the experiments under clean conditions, which might have disrupted the gut microbiome thereby resulting in different results. Most experimental results and human obese specimen results indicated that LNK was involved in obesity-induced adipose dysfunction. The metabolic system is complex and obesity-induced insulin resistance may be the result of a combination of causes.

**Figure 6:**
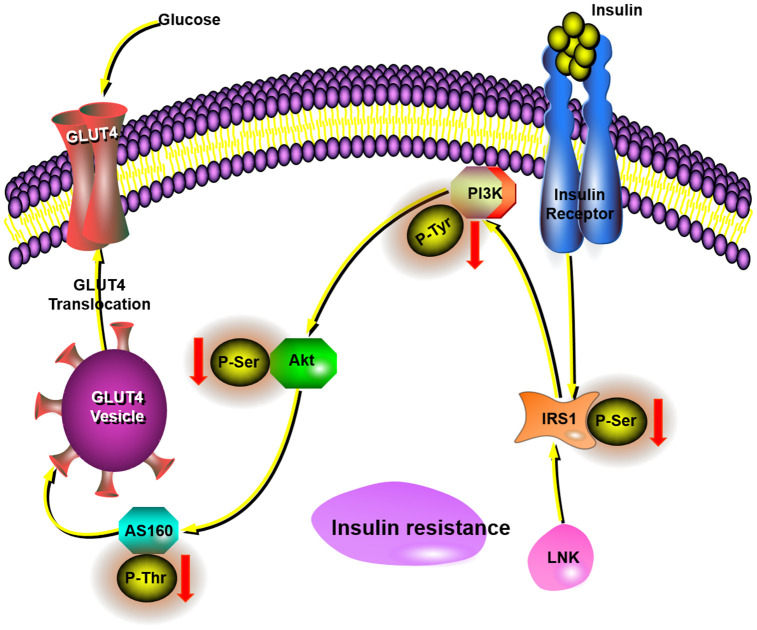
**This schematic diagram showed LNK mediated insulin-stimulated glucose transportation through the IRS1-PI3K-Akt-AS160 pathway in adipocytes.**

In conclusion, LNK deficiency has direct effects on insulin signaling and glucose translocation, which all collectively leads to increase glucose uptake in adipose tissue and improved insulin sensitivity in obesity. Our data suggests that LNK inhibits the glucose translocation of adipose tissue through the PI3K-Akt-AS160 pathway in obesity-induced insulin resistance (Figure 6). As such, these findings amplify the underlying mechanisms connecting glucose translocation and insulin resistance. Although the contribution of glucose translocation to insulin resistance is reasonably well established, therapeutic attempts to correct this have not had adequate success in clinical studies. Based on our findings with LNK, one can raise the possibility that previous therapeutic attempts aimed to ameliorate insulin resistance. These studies indicate that the inhibition of LNK could be a future targeted therapy for the prevention of insulin resistance.

## MATERIALS AND METHODS

### Human samples

The study protocol was approved by the Research Ethics Board of Sun Yat-sen memorial hospital of Sun Yat-sen University and the first affiliated hospital of Guangzhou Medical University. All participants signed a consent form before the surgery. Seventy-two patients were included from January 1, 2017 to June 30, 2018. Adipose tissue samples were obtained from 31 patients who received laparoscopic surgery for fallopian tube or pelvic disorders in the Gynecologic department of Sun Yat-sen Memorial Hospital of Sun Yat-sen University, and 41 patients received abdominal surgery for gastrointestinal tumors or extreme obesity in the First Affiliated Hospital of Guangzhou Medical University. Individuals with a history of cardiovascular or cerebrovascular disease, severe hepatic disease, severe renal disease, autoimmune diseases, immunodeficiency, or individuals receiving immunosuppressive treatment were excluded from this study. Omental adipose tissue (4–6 g) from the peripheral portion of the *omentum majus* was collected from the patients during surgery. Samples were immediately put in liquid nitrogen for further experiments.

### Animals

All of the experimental procedures were approved by the Committee for Animal Research of Sun Yat-sen University and were in accordance with the National Institutes of Health (NIH) Guide for the Care and Use of Laboratory Animals. Male C57BL/6 wild-type mice were purchased from the animal research center of Sun Yat-sen University. Whole-body LNK-deficient on C57BL/6 genetic background mice were created by CRISPR/Cas-mediated genome engineering by Cyagen Biosciences Inc. The mouse Sh2b3 gene (GenBank accession number: NM_001306127.1; Ensembl: ENSMUSG00000042594) is located on mouse chromosome 5. Exon 1 to exon 3 were selected as target sites. Cas9 mRNA and gRNA generated by *in vitro* transcription were then injected into fertilized eggs for knock-out mouse production. The founders were genotyped by PCR followed by DNA sequencing analysis. The positive founders were bred to the next generation (F1) and subsequently genotyped by PCR and DNA sequencing analysis. (Supplementary Figure 1).

Mice were housed in numbers of four to six per cage with standard laboratory conditions (12 h light:12 h darkness cycle) and free access to rodent feed and water. Mice (4-5 weeks old) were fed with a high-fat diet (HFD, 60% calories from fat, D12492; Research Diets Inc.) or normal chow diet (NCD, 10% calories from fat, D12450; Research Diets Inc.) for 0, 2, 4, 8 or 16 weeks (n=6 or 7 per group). At the end of the diet intervention, mice were anesthetized with sodium pentobarbital (Sigma-Aldrich) at 50 mg/kg (intraperitoneal, *i.p.*). Blood, liver, epididymal fat pads, perirenal fat and other visceral adipose tissue were collected.

### Measurements of metabolic parameters

Food intake and body weight were measured once a week. Briefly, for the glucose tolerance test (GTT), mice which were fasted overnight were injected (*i.p.*) with D-glucose (1 g/kg body weight; Sigma-Aldrich) after 12 h fasting. For the insulin tolerance test (ITT), mice were injected (*i.p.*) with insulin (0.75 units/kg body weight; Novolin R, Novo Nordisk, Copenhagen, Denmark) after 6 h fasting. Glucose concentrations were measured in tail venous blood using an automated glucometer (Roche Diagnostics) up to 90 min or 120 min after injection.

Fasting insulin was measured by the insulin ELISA kit (Cusabio, Wuhan, China). Liver concentrations of triglyceride (TG) was measured by the triglyceride assay kit (Nanjing Jiancheng Bioengineering Institute, Nanjing, China). Serum concentrations of total cholesterol (TC), triglyceride (TG), high density lipoprotein (HDL), low density lipoprotein (LDL), apolipoprotein E (ApoE), FFA and hsCRP were measured by enzymatic method using an automatic biochemical analyzer (Beckman AU5800, USA).

### Magnetic resonance imaging (MRI) of lower abdominal visceral adipose tissue (VAT)

The volume of mouse abdominal VAT was measured *in vivo* via MRI (Philips Ingenia 3.0T, Netherlands) equipped with a mouse 8-channel coil. After 16 weeks of HFD feeding, mice were anesthetized with pentobarbital (50 mg/kg) and fixed in an entirely stretched supine position to minimize posing error. MRI T1WI-TSE-TRA, T1WI-TSE-COR and T1WI-HR-mDIXON-COR sequences were conducted to image the abdominal region. The scan was constructed for the whole abdominal region, which begins from the diaphragm to the pelvic floor. The volume of total abdominal adipose tissue was calculated using the regions of interest (ROI) by Image-Pro Plus 6.0 (Media Cybernetics, Inc., Singapore).

### Histological and immunohistochemical staining

The mouse epididymal fat and liver tissue were fixed in 4% paraformaldehyde (Sigma-Aldrich), embedded in paraffin and sectioned at 10 μm. Paraffin-embedded tissue sections were stained with H&E. Immunohistochemistry was then performed to detect the location of LNK and the expression of GLUT4 in adipose tissue. Briefly, five randomly selected sections in each sample were exposed to primary antibodies (LNK, 1:50, Abcam; GLUT4, 1:200, Servicebio) or goat serum as the negative control at 4°C overnight, followed by incubation with corresponding secondary antibodies. Histological images were captured using a Nikon Instruments Eclipse Ni-U microscope (Japan) with plan×4, ×10, ×20, or ×40 objective lenses.

### Cell culture

The primary preadipocytes were firstly isolated from the mouse epididymal fat tissue. The adipose tissue was minced in PBS with 0.5% BSA, and then incubated with digestion buffer [DMEM, 12.5 mM HEPES (pH 7.4), 2% BSA, and 10 mg collagenase type I (Sigma)] in a 37°C shaking water bath (115 r/min) for 40 min. The digested tissue was filtered through a 250-μM nylon sieve and centrifuged at 500×g for 5 min. The pellet containing preadipocytes was incubated with an erythrocyte-lysing buffer, washed and spun at 300×g for 5 min to obtain the preadipocytes. Primary preadipocytes were cultured in DMEM medium (GIBCO) containing 10% fetal bovine serum (FBS, GIBCO) and 1% penicillin/streptomycin at 37°C in a humidified atmosphere of 5% CO_2_. Differentiation of preadipocytes to adipocytes was induced by an adipogenic cocktail. Briefly, after primary preadipocytes reached confluency, the cells were incubated in differentiation medium (DM) containing 1 μM DEX (Sigma), 0.5 mM IBMX (Sigma), and 10 μg/mL insulin (BI) in DMEM with 10% FBS for 6 days. The cell culture medium was changed to post-DM containing 10 μg/mL insulin in DMEM with 10% FBS and post-DM was freshly replaced every 48 h. Differentiated cells were used when at least 95% of the cells showed an adipocyte phenotype by accumulation of lipid droplets. Differentiated cells were stimulated by LY294002, a PI3K inhibitor (Absin, Shanghai, China, 50 μM) for 30 min or insulin (Biological Industries, Israel, 10 μM) for 30 min.

### Insulin stimulation studies and glucose uptake assays

For insulin stimulation studies, cells were washed twice with warm PBS and serum starved in serum-free DMEM containing 0.5% bovine serum albumin 16 h before insulin stimulation. On the day of the assay, the medium was replaced with 100 μL of serum-free DMEM with or without 50 μM LY294002 and incubate for 30 min at 37°C in 5% CO_2_. 10 μL of serum-free DMEM with or without 10 μM insulin was added and incubated for 30 min. Glucose uptake was assessed on Day-10 of differentiation in 96-well plates using the Glucose Uptake-Glo™ Assay (Promega, WI, USA). The medium was removed and washed with 100 μL of PBS if glucose was present. 50 μL of the prepared 1 mM 2DG was added per well, shaken briefly, and incubated for 10 min at room temperature. 25 μL of Stop Buffer was added and shaken briefly. 25 μL of Neutralization Buffer was then added and shaken briefly. Subsequently, 100 μL of 2DG6P Detection Reagent was added and shaken briefly. Incubation was performed for 30 min at room temperature and the luminescence was recorded with 0.3-1 s integration on a luminometer.

### Quantitative real-time PCR

Total RNA was extracted from tissues or purified cells using RNAiso Plus reagent (9109, TAKARA, Japan) according to the manufacturer’s protocols. RNA was reverse-transcribed using the Primescriptor RT Master Mix (RR036A, TAKARA, Japan). Quantitative RT-PCR was performed using the TB Green Premix Ex Taq II (RR820A, TAKARA, Japan). Primer sequences are provided in Supplementary Table 1. As for the calculation, we selected one of the patients as a reference, and the 2^-ΔCT^ value of all other patients was divided by the 2^-ΔCT^ value of this patient. "1.0" represent for the mRNA value of the selected patient.

### Western blotting

Tissue or cells were lysed using RIPA lysis buffer (Beyotime, Nanjing, China) containing protease inhibitors and phosphatase inhibitors (Complete; Roche). Equal amounts of protein (quantified by BCA protein assays, Thermo Scientific) in each sample were analyzed by SDS-PAGE and immunoblotting. Protein samples were stored at -80°C before the experiments. Samples were separated on 10% SDS polyacrylamide gels and then transferred to polyvinylidene difluoride membrane (Bio-Rad). The membrane was incubated with corresponding primary antibodies overnight. On the next day, the membrane was further incubated with appropriate horseradish peroxidase-conjugated secondary antibodies for 1 h. The bands were detected by enhanced chemiluminescence (ECL) detection system (Affinity Biosciences, OH, USA). Invitrogen iBright CL1000 Imaging System was used to expose the bands. The primary antibodies are provided in Supplementary Table 2. Densitometric analysis was performed with ImageJ software (NIH, Bethesda, MD).

### Statistical analysis

All data were presented as mean ± SEM. Statistical analyses were performed using Student’s *t*-test or one-way ANOVA followed by Bonferroni post-hoc test. Pearson correlation analysis was used to determine the relationship of LNK mRNA expression in adipose tissue and metabolic indexes in patients. A *P*-value <0.05 was considered to be statistically significant. All statistical analyses were performed using SPSS 22.0 (SPSS, Inc., Chicago, IL, USA). Figures were created with GraphPad Prism 8.0 (San Diego, CA, USA).

## Supplementary Material

Supplementary Figures

Supplementary Tables
