# Supplementary material for: LNK deficiency decreases obesity-induced insulin resistance by regulating GLUT4 through the PI3K-Akt-AS160 pathway in adipose tissue
Source: Aging (Albany NY). 2020 Sep 10;12(17):17150–66. doi: 10.18632/aging.103658 (PMC7521507; doi:10.18632/aging.103658)
Supplement: Supplementary Figures [file aging-12-103658-s002..pdf]

SUPPLEMENTARY FIGURES

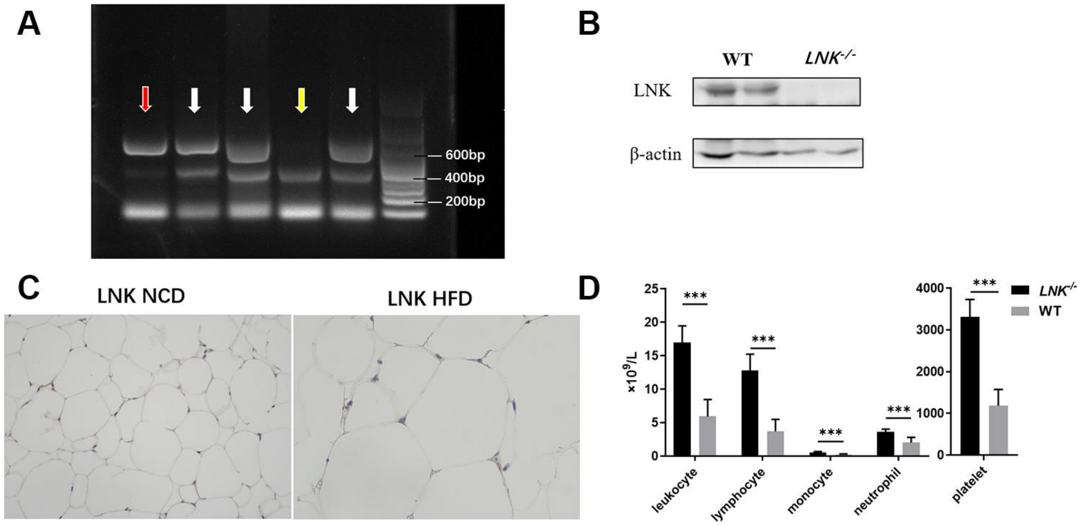

Supplementary Figure 1. Genotype identification of LNK knockout mice. (**A**) Homozygote (red arrow), heterozygote (white arrow), WT (yellow arrow) and marker band were shown. Total DNA was isolated from mice tail between 8-14 days of age, and PCR was performed using one Step Mouse GenoTyping Kit (PD101-01, Vazyme Biotech, Nanjing, China). Primer sequences: (left of the marker) Mouse Sh2b3-F: 5'-CCAATGTGGCGATAGAGAAAAGGTAAAG-3', (right of the marker) Mouse Sh2b3-Wt/He-F: 5'-TGTCATTTCATCTTTTCTCTGTGGTCC-3', Mouse Sh2b3-R: 5'-CAAACCTCAGGTCAGCCTCTACATAGC-3'. Product Size: delete: 610 bp, WT: 459 bp. Annealing Temp: 60°C. (**B**) LNK expression in WT and *LNK*$^{-/-}$ mice was examined by Western blot analysis using an antibody to LNK. Protein extracts were prepared from the ears. For a ~10 mg piece of ear tissue, add ~200 µL of ice cold strong RIPA lysis buffer rapidly to the EP tube, homogenize with an electric homogenizer. Centrifuge for 15 min at 12,000 rpm at 4°C in a microcentrifuge after incubation on ice for 30 min. Gently remove the tubes from the centrifuge and place on ice, aspirate the supernatant and place in a fresh tube kept on ice; discard the pellet. The protein concentration is 1.2 mg/mL. Load 30 µg of protein into the wells of the SDS-PAGE gel and run the gel. (**C**) The immumohistochemical staining for LNK protein in adipose tissues of *LNK*$^{-/-}$ mice with NCD or HFD feeding for 16 weeks was shown. (**D**) Peripheral blood routine examination of *LNK*$^{-/-}$ (open symbols, n = 6) and WT (filled symbols, n = 6) male mice between 6-8 weeks of age were measured. Data were shown as mean ± S.D. Statistical analysis was performed by Student's *t*-test. *p<0.05, **p<0.01, ***p<0.001.

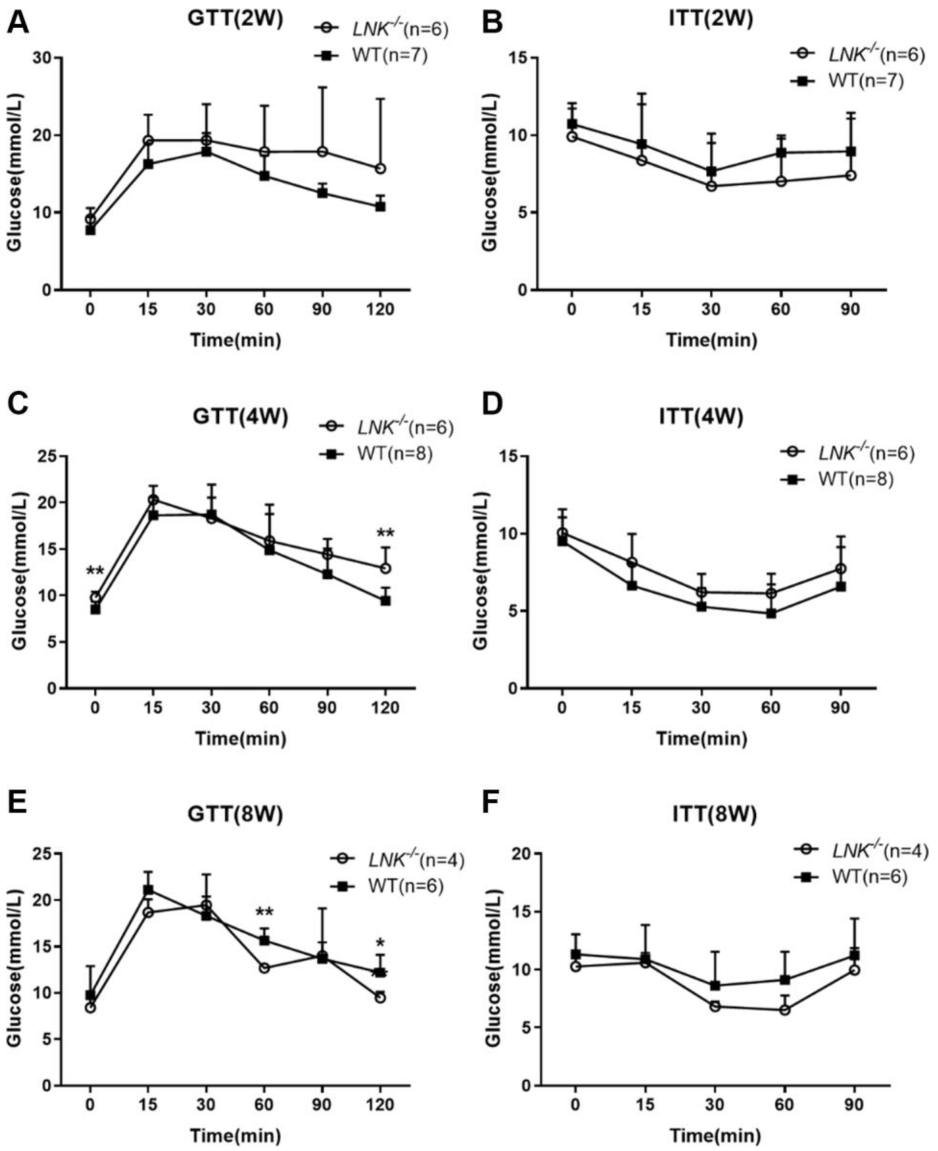

Supplementary Figure 2. LNK relieved impaired glucose tolerance in HFD feeding mice for 4 weeks, but not at 2 and 8 weeks. GTTs (**A**, **C** and **E**) and ITTs (**B**, **D** and **F**) in WT (filled symbols, n = 4-6) or *LNK*$^{-/-}$ mice (open symbols, n = 6-8) with HFD feeding for 2, 4 and 8 weeks were measured. Data were shown as mean ± S.D. Statistical analysis was performed by Student's *t*-test. *p<0.05, **p<0.01.
