# Supplementary material for: LNK deficiency decreases obesity-induced insulin resistance by regulating GLUT4 through the PI3K-Akt-AS160 pathway in adipose tissue
Source: Aging (Albany NY). 2020 Sep 10;12(17):17150–66. doi: 10.18632/aging.103658 (PMC7521507; doi:10.18632/aging.103658)
Supplement: Supplementary Tables [file aging-12-103658-s001..pdf]

SUPPLEMENTARY TABLES

Supplementary Table 1. Primer sequences used in Quantitative RT-PCR.

Gene	Forward Primer	Reverse Primer
ATGL	GTGCCAACATTATTGAGGTG	GAAACACGAGTCAGGGAGAT
ATF6	TCGCCTTTTAGTCCGGTTCTT	GGCTCCATAGGTCTGACTCC
β-actin	AGCCATGTACGTAGCCATCC	GCTGTGGTGGTGAAGCTGTA
BIP	GACTGCTGAGGCGTATTTGG	AGCATCTTTGGTTGCTTGTCG
CD206	TTGATTGCCACTTTTGAAGATACC	TGAGACCTACCACCAGTGTAGCA
C/EBP α	CTGGGTGAGTTCATGGAGAATG	CCTACCACATGGCTAACTCACAAC
C/EBP β	TGACGCAACACACGTGTAACTG	ATCAACAACCCCGCAGGAA
CHOP	AAGCCTGGTATGAGGATCTGC	TTCCTGGGGATGAGATATAGGTG
CREB	TTGAGGTCCGGTGAGATTCC	TGTGAGTGCTGGAGTAAAACAGTCA
FOXO1	ATGCTCAATCCAGAGGGAGG	ACTCGCAGGCCACTTAGAAAA
G6pc2	CAGGAGGACTACCGGACTTAC	TCAACTGAAACCAAAGTGGGAA
GLUT4-mouse	ACACTGGTCCTAGCTGTATTCT	CCAGCCACGTTGCATTGTA
GLUT4-human	ATCCTTGGACGATTCCTCATTGG	CAGGTGAGTGGGAGCAATCT
HSL	GACTCACCGCTGACTTCC	TGTCTCGTTGCGTTTGTAG
LNK-mouse	CCAATGTGGCGATAGAGAAAAGGTAAAG	CAAACCTCAGGTCAGCCTCTACATAGC
LNK-human	AATAGGAGGGTGGCAAGAACAG	TCCCCTAGCCCCAGTTAATTTAA
MCP-1	CTTCCTCCACCACCATGCA	AGCCGGCAACTGTGAACAG
PEPCK	AGCATTCAACGCCAGGTTC	CGAGTCTGTCAGTTCAATACCAA
PPAR γ	GCCCACCAACTTCGGAATC	TGCGAGTGGTCTTCCATCAC
SREBP-1	CCAGAGGGTGAGCCTGACAA	AGCCTCTGCAATTTCCAGATCT
Trem2	CTGGAACCGTCACCATCACTC	CGAAACTCGATGACTCCTCGG

ATGL, adipose triglyceride lipase; ATF6, activating transcription factor 6; BIP, binding immunoglobulin protein; CD206, cluster of differentiation 206; C/EBP α, CCAAT/enhancer binding protein alpha; C/EBP β, CCAAT/enhancer binding protein beta; CHOP, C/EBP homologous protein; CREB, cAMP-response element binding protein; FOXO1, forkhead box class O1; G6pc2, glucose-6-phosphatase 2; GLUT4, glucose transporter 4; HSL, hormone-sensitive lipase; MCP-1, monocyte chemotactic protein-1; PEPCK, phosphoenolpyruvate carboxykinase; PPAR γ, peroxisome proliferator-activated receptor gamma; SREBP-1, sterol-regulator element-binding protein-1; Trem2, triggering receptor expressed on myeloid cells 2.

Supplementary Table 2. Antibodies used in Western blot.

Protein	Company	Product Code	Dilution	Application
Akt	Cell Signaling Technology	4685	1:1000	WB
Akt (Ser473)	Cell Signaling Technology	4060	1:1000	WB
AS160 (Thr642)	Cell Signaling Technology	8881	1:1000	WB
β-actin	Proteintech	66009-1-Ig	1:1000	WB
GLUT4	Cell Signaling Technology	2213S	1:1000	WB
GLUT4	Servicebio	GB11244	1:500	IHC
IRS1	Cell Signaling Technology	2382	1:1000	WB
IRS1 (Ser307)	Abclonal	AP0552	1:1000	WB
LNK	Santa Cruz Biotechnology	sc-393709	1:1000	WB
LNK	Abcam	ab 244278	1:50	IHC
PI3K	Cell Signaling Technology	4292	1:1000	WB
PI3K (p85(Tyr458)/ p55(Tyr199))	Cell Signaling Technology	4228	1:1000	WB

AS160, Akt substrate of 160 kDa; GLUT4, glucose transporter 4; IRS1, insulin receptor substrate 1; PI3K, phosphatidylinositol 3-kinase.
